# A case report of falsely elevated high-sensitivity cardiac troponin I due to macro-troponin I

**DOI:** 10.1016/j.plabm.2026.e00546

**Published:** 2026-07-08

**Authors:** Yinfu Sun, Jiawen Shang, Weihong Qin, Ziyuan Peng, Miaojun Lia, Jingzhe Wang

**Affiliations:** Shenzhen Key Laboratory of Medical Laboratory and Molecular Diagnostics, The First Affiliated Hospital of Shenzhen University, Shenzhen Second People's Hospital, Shenzhen Second People's Hospital Longhua Hospital, Shenzhen, 518035, China

**Keywords:** Hs-cTnI interferences, Macro-troponin I, Cardiac biomarkers

## Abstract

This article reports a clinical case of falsely and significantly elevated high-sensitivity cardiac troponin I (hs-cTnI) levels caused by “Macro-Troponin I". A 54-year-old male patient was admitted with a diagnosis of acute non-ST-segment elevation myocardial infarction (NSTEMI). While his hs-cTnI levels rose sharply during the acute phase and subsequently persisted at an extremely high plateau level (>27404 ng/L) without decline for several days, his myoglobin (MYO) and creatine kinase-MB (CK-MB) levels followed the expected pattern of initial elevation followed by resolution. This discrepancy raised suspicion regarding the validity of the hs-cTnI results. Subsequent systematic laboratory verification, including multi-platform assay comparison revealing a vast discrepancy between hs-cTnI and high-sensitivity cardiac troponin T (hs-cTnT) results, non-linear recovery upon serial dilution, and a marked decrease in hs-cTnI to normal levels after polyethylene glycol (PEG) precipitation, confirmed interference from a precipitable macromolecular complex. Furthermore, the interference could not be eliminated by various common heterophilic antibody blocking agents.

Based on laboratory results, we confirmed the presence of a macromolecular interference in the patient's plasma, which was highly likely to be macro cardiac troponin I due to its ultra-long half-life.Regarding the origin of macro cardiac troponin I, current research indicates that anti-troponin antibodies exist, which rapidly form immune complexes upon exposure to troponin antigens, thereby interfering with experimental data.Through this case, we demonstrated the presence of macro-complexes interference. The patient ultimately received correct diagnosis and management due to timely recognition of this interference.

## 1 Introduction

Cardiac Troponin I (cTnI) is not normally present in​ skeletal or smooth muscle, its elevation strongly indicates​ myocardial cell injury, with very few false positives [1]. Therefore, upon detecting elevated cTnI, clinicians can largely rule out “non-cardiac causes" and confirm myocardial damage. It rises within 3-4 hours of onset, offering high sensitivity about 2 hours earlier than CK-MB, and remains elevated for over 10 days, allowing detection even in patients with delayed presentation (>12 hours), making it suitable for “difficult chest pain" cases in the emergency room laboratory [2,3].

In patients with coronary artery disease (CAD), plasma high-sensitivity cardiac troponin I (hs-cTnI) levels show a significant positive correlation with the severity of coronary lesions (Gensini score), suggesting that higher hs-cTnI concentrations indicate more severe coronary disease [4]. In risk stratification for suspected acute coronary syndrome (ACS), elevated hs-cTnI is positively associated with the presence of CAD, indicating that it may reflect the severity of coronary disease [5]. Clinically, pre-angiography hs-cTnI levels can serve as a screening tool. Low levels might suggest deferring angiography or opting for non-invasive tests (like CT angiography), while high levels indicate the need for prompt diagnostic angiography. Combined with Gensini score or angiographic findings, hs-cTnI aids in building precise risk stratification models to guide treatment intensity.

However, in routine clinical testing, hs-cTnI is subject to interference from multiple factors that may cause falsely elevated or decreased results. Pre-analytically, lipemia, hemolysis, and icterus in specimens may affect cTnI measurements to varying degrees. More commonly and with greater clinical significance are endogenous interfering factors—related to the patient's immune status, abnormal proteins, and drug metabolism—including: heterophilic antibodies, rheumatoid factor, autoantibodies, macro-immune complexes, phosphorylation or protein hydrolysis, cross-reactivity with skeletal muscle troponin, and specific metabolic or disease states [6,7]. As reported in the literature, these interfering factors may lead to erroneous test results, thereby negatively impacting clinical diagnosis and treatment [8]. Therefore, timely identification and correction of such results is of particular importance.

## 2 Case description

A 54-year-old male was admitted to the hospital due to chest pain persisting for over 13 hours. The patient experienced sudden, unexplained epigastric colicky pain approximately 13 hours prior to admission. The pain was persistent, progressive, and associated with chest tightness, radiation to the left shoulder and left maxillary area, cold sweats, and palpitations. He subsequently presented to our emergency department. Arrival at ER and performed ECG, ECG results indicated: 1. Sinus rhythm, 2. ST-segment depression in leads aVL and V4-V6, 3. T-wave inversion. Troponin T(Point-of-Care Testing) measured was <0.010 μg/L. Hs-cTnI was elevated at 144 ng/L. Based on assessment of Cardiology, a diagnosis of acute NSTEMI was considered. The patient was administered Aspirin Enteric-Coated Tablets and Clopidogrel orally. On September 26th at 08:04, a repeat hs-cTnI level was significantly elevated at 15236 ng/L. Following re-evaluation and after loading with antiplatelet agents, the patient was transferred directly to the Digital Subtraction Angiography laboratory (DSA lab) for emergency coronary angiography, bypassing the Coronary Care Unit (CCU).

### 2.1 Examinations

Cardiac Markers (2025-09-25):Hs-cTnI 144 ng/L ↑, Myoglobin 23.4 ng/ml, CK-MB 2.01 ng/ml;

Ultrasound: Showed occasional arrhythmia during exam, slightly enlarged left atrium, dilated ascending aorta, aortic valve degeneration with mild regurgitation, mild mitral and tricuspid regurgitation, impaired left ventricular diastolic function, preserved systolic function (EF: 63%).

ECG: Sinus rhythm, ST-segment depression in aVL and V4-V6, T-wave inversion.

Repeat Cardiac Markers (2025-09-26,00:00): Hs-cTnI 271 ng/L ↑, Myoglobin 223.0 ng/ml ↑, CK-MB 7.36 ng/ml ↑.

Repeat Cardiac Markers (2025-09-26, 08:04): Hs-cTnI 15236 ng/L ↑↑↑, Myoglobin 138.8 ng/ml ↑, CK-MB 125.05 ng/ml ↑.

Coronary Angiography: Left dominant circulation. Left main: normal. Left Anterior Descending (LAD): large vessel, diffuse irregularity, 30% diffuse stenosis proximally, significant tortuosity in proximal-mid segment, 30-50% diffuse stenosis in proximal-mid segment, 40-50% segmental stenosis in mid-distal segment, TIMI flow III. Left Circumflex (LCx): large vessel, diffuse irregularity, 20% stenosis proximally, 30% stenosis mid, 50-60% stenosis mid-distal, 70% segmental stenosis at the distal bifurcation with the obtuse marginal branch, TIMI flow III. Right Coronary Artery (RCA): relatively small, diffuse irregularity, localized ectasia proximally, 30-50% diffuse stenosis in proximal-mid to mid segment, TIMI flow III.

## 3 Laboratory evaluation

### 3.1 Persistently elevated hs-cTnI levels

**Fig. 1:**
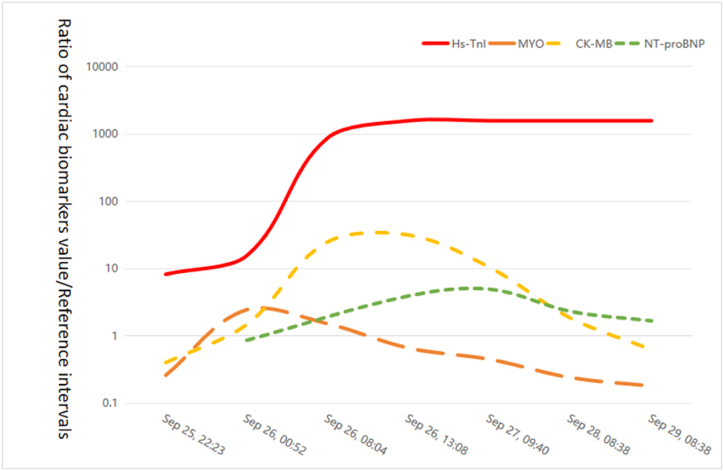
Serial monitoring of cardiac biomarkers post-admission.

Serial monitoring of cardiac biomarkers post-admission revealed persistently elevated hs-cTnI(Specific values are shown in Supplementary Table 1). As shown in Fig. 1, hs-cTnI rose sharply from 144 ng/L (Sep 25, 22:23) to 15236 ng/L (Sep 26, 08:04), and remained at a very high plateau (>27404 ng/L) from Sep 26, 13:08 until at least Sep 29. In stark contrast, MYO and CK-MB followed the typical pattern of rise and fall. NT-proBNP also showed corresponding changes. This discordance—persistently extremely high hs-cTnI while other markers normalized—was the initial key clue suggesting a potential assay anomaly.

### 3.2 Multi-platform assay discrepancy

To verify reliability, the patient's plasma was tested on different platforms. Table 1 shows that for the same sample, hs-cTnI was 28107 ng/L on the Beckman platform and even higher at 38405 ng/L on the Abbott platform. However, hs-cTnT measured on the Roche platform was only 74.6 ng/L. This significant discrepancy between hs-cTnI results across platforms and the orders-of-magnitude difference from the hs-cTnT result strongly suggested specific interference in the Beckman and Abbott hs-cTnI assays (Table 1).

**Table 1:** Multi-platform troponin assay results.

Assay	Result	99th percentile URL	fold increase	imprecision at 99th percentile
Beckman DXI800 hs-cTnI	28107 ng/l	17.5ng/l	1606.11	3.7%
Abbott i2000 hs-cTnI	38405 ng/l	26.2ng/l	1465.84	4.0%
Roche cobas801 hs-cTnT	74.6 ng/l	14ng/l	5.33	5%

### 3.3 Non-linear dilution and PEG precipitation

Serial dilution studies were performed. As per Table 2, the neat plasma hs-cTnI was >27404 ng/L. With 2-fold, 4-fold, and 8-fold dilution, measured concentrations were 28107, 26692, and 23624 ng/L respectively, indicating non-linear recovery—a classic sign of interference. Subsequent treatment with PEG to precipitate macromolecules resulted in a dramatic decrease of hs-cTnI to 148 ng/L (within normal limits), a value concordant with the hs-cTnT level. This confirmed the presence of a PEG-precipitable macromolecule causing falsely elevated hs-cTnI. The recovery rate was up to 99.5% way over the 20% cutoff value [9] (Table 2).

**Table 2:** Hs-cTnI serial dilution and PEG precipitation.

Sample Treatment	Beckman Hs-cTnI (ng/L)	Recovery
Neat	>27404	/
2-fold Dilution	28107	/
4-fold Dilution	26692	5%
8-fold Dilution	23624	16%
PEG Precipitation	148	99.5%

### 3.4 Lack of effect from heterophilic blocking agents

Various blocking agents were used. As shown in Table 3, treatment with HBR-1 (heterophilic antibody blocker), animal IgGs (goat, mouse, rabbit, bovine, sheep), or commercial blockers (POLY-MAK, AP-P001) resulted in no response effect (recovery rate greater than 20%) compared to the neat sample [10]. This lack of response to specific blockers further ruled out common interferents like heterophilic antibodies or human anti-animal antibodies, supporting the interference with macro-complexes (Table 3).

**Table 3:** Effect of blocking agents on hs-cTnI.

	Raw Result (ng/L)	Blocking Result(Blocking Result = Raw Result/0.9. 0.9 is dilution factor.) (ng/L)
Neat Sample	>27404	>27404
Neat Control (PBS)	>27404	>27404
HBR-1	>27404	>27404
POLY-MAK	>27404	>27404
Goat IgG	>27404	>27404
Mouse IgG	>27404	>27404
Rabbit IgG	>27404	>27404
Bovine IgG	>27404	>27404
Sheep IgG	23910	26570
AP-P001	23020	25580

## 4 Discussion

In this patient, the abnormally persistent, extremely high hs-cTnI level (>27404 ng/L) after the acute phase should have predicted extremely severe obstructive coronary disease [8,11]. However, coronary angiography revealed the most severe stenosis was only “70% segmental stenosis in the distal segment," with the rest mostly being 30%-50% diffuse stenoses, and all vessels had TIMI III (normal) flow. This degree of disease was completely mismatched with the severe myocardial injury implied by the persistently high hs-cTnI, indicating false elevation of hs-cTnI.

Based on the literature, causes of falsely elevated hs-cTnI include heterophilic antibodies, which are weak antibodies against undefined antigens that can cross-react with animal-derived immunoglobulins in assay reagents, forming a “bridge" between capture and detection antibodies and causing false-positive signals. This interference may lead to chronic, baseline elevation, sometimes with dynamic changes [12], and patient history might involve animal contact, recent transfusion, vaccination, or autoimmune diseases; however, the use of heterophilic blocking agent HBR-1 and multiple animal antibodies showed no effect, ruling out this interference. Rheumatoid factor (RF) is an autoantibody that interferes similarly by binding the Fc portion of assay antibodies, but screening the patient's plasma for RF was negative, eliminating RF interference [13]. Alkaline phosphatase (ALP) interference is specific to assays using ALP as the signal enzyme, such as some Beckman platforms, and since the patient had a high result on the Abbott platform (which does not use ALP) and AP-P001 blocking agent had no effect, ALP interference was ruled out.

After excluding multiple common pre-analytical and analytical interfering factors, the source of discrepancy remained unidentified. A retrospective review of the patient's clinical course revealed an initial alignment between the diagnosis of NSTEMI and elevated hs-cTnI levels. However, on September 26 at 08:04, the hs-cTnI value surged dramatically.Despite the critical laboratory alert triggering an emergency coronary angiography, the procedure revealed no coronary artery occlusion, and the patient denied any more severe symptoms. This finding was inconsistent with the markedly elevated hs-cTnI results. Furthermore, the persistently high hs-cTnI levels contradicted the patient's favorable clinical trajectory and symptomatic improvement, in the same time, other cardiac biomarkers showed normal fluctuations.The polyethylene glycol (PEG) precipitation test confirmed the presence of macro-complexes. Consequently, we hypothesized a post-release interference affecting hs-cTnI specifically, the formation of a long-lasting, high-reactivity complex. Based on literature reviews [14], this presentation is highly suggestive of macro-troponin I (a high molecular weight complex). To validate the persistence of this interference, post-discharge follow-up were taken on October 5. While other cardiac biomarkers (Myoglobin: 16.3 ng/mL; CK-MB: 2.05 ng/mL) had returned to negative levels, hs-cTnI remained markedly elevated at >27,404 ng/L. This prolonged half-life strongly supports the hypothesis that macro-troponin I is the underlying cause of the discordance, and it would chronically affect clinical diagnosis and therapeutic decision-making [10].

Based on increasing case reports over the past decade, macrotroponin has been increasingly recognized. It is a large molecular immune complex formed between troponin and circulating autoantibodies [15]. With a significantly longer half-life than free troponin, macrotroponin causes persistent, stable “plateau-like" false elevations that often contradict clinical symptoms. Notably, reported cases occurred following some degree of myocardial injury [16]. This observation suggests that troponin, upon release, may expose antigenic epitopes that become targets for antibody recognition. There is literature summarized that anti-troponin antibodies are widely present in the general population. However, the mechanisms behind their formation and their potential impact on patient outcomes remain unclear, warranting further investigation.

This paper reports a case of false hs-cTnI elevation induced by macro-cTnI: thanks to the rich clinical experience of physicians and the timely and comprehensive supplementary tests conducted by the laboratory, the actual cTnI level was finally identified. The patient's condition improved after targeted treatment and he was discharged smoothly.

## CRediT authorship contribution statement

**Yinfu Sun:** Investigation, Methodology, Writing – original draft. **Jiawen Shang:** Methodology, Writing – original draft, Writing – review & editing. **Weihong Qin:** Formal analysis, Investigation, Writing – review & editing. **Ziyuan Peng:** Formal analysis, Resources. **Miaojun Lia:** Project administration, Supervision. **Jingzhe Wang:** Funding acquisition, Writing – review & editing.

## Declaration of competing interest

The authors declare that they have no known competing financial interests or personal relationships that could have appeared to influence the work reported in this paper.

## Data Availability

No data was used for the research described in the article.
