# Supplementary material for: A case report of falsely elevated high-sensitivity cardiac troponin I due to macro-troponin I
Source: Pract Lab Med. 2026 Jul 8;51:e00546. doi: 10.1016/j.plabm.2026.e00546 (PMC13380090; doi:10.1016/j.plabm.2026.e00546)
Supplement: Multimedia component 1 [file mmc1.docx]

| Date/Time | Hs-TnI | MYO | CK-MB | NT-proBNP |
| --- | --- | --- | --- | --- |
| Sep 25, 22:23 | 8.23 | 0.26 | 0.402 | / |
| Sep 26, 00:52 | 15.49 | 2.45 | 1.472 | 0.86 |
| Sep 26, 08:04 | 870.63 | 1.52 | 25.01 | / |
| Sep 26, 13:08 | 1565.94 | 0.66 | 31.548 | 3.93 |
| Sep 27, 09:40 | 1565.94 | 0.45 | 10.392 | 4.95 |
| Sep 28, 08:38 | 1565.94 | 0.24 | 1.84 | 2.31 |
| Sep 29, 08:38 | 1565.94 | 0.18 | 0.626 | 1.68 |

**Supplementary Table 1**Specific values of serial monitoring of cardiac biomarkers post-admission

| Assay | Reference intervals |
| --- | --- |
| Beckman DXI800 hs-cTnI | 0-17.5ng/l |
| Beckman DXI800 Myo | Male：15.2-91.2ng/ml  Female：11.1-57.5ng/m |
| Beckman DXI800 CK-MB | 0.5-5.0ng/ml |
| Beckman DXI800 Ntpro-BNP | Male（18-49）：0-87.13ng/L  Female（18-49）：0-139.64ng/L  Male（50-75）：0-156.68ng/L  Female（50-75）：0-182.58ng/L |

**Supplementary Table 2** Reference intervals for the cardiac biomarkers
